# REDBot: Natural language process methods for clinical copy number variation reporting in prenatal and products of conception diagnosis

**DOI:** 10.1002/mgg3.1488

**Published:** 2020-09-22

**Authors:** Mengmeng Liu, Yunshan Zhong, Hongqian Liu, Desheng Liang, Erhong Liu, Yu Zhang, Feng Tian, Qiaowei Liang, David S. Cram, Hua Wang, Lingqian Wu, Fuli Yu

**Affiliations:** ^1^ Berry Genomics Corporation Beijing China; ^2^ Department of Obstetrics and Gynecology West China Second University Hospital Sichuan University Chengdu; ^3^ Center for Medical Genetics School of Life Sciences Central South University Changsha China; ^4^ Hunan Jiahui Genetics Hospital Changsha China; ^5^ Hunan Provincial Maternal and Child Health Care Hospital Changsha China; ^6^ Human Genome Sequencing Center Department of Molecular and Human Genetics Baylor College of Medicine Houston TX USA

## Abstract

**Background:**

Current copy number variation (CNV) identification methods have rapidly become mature. However, the postdetection processes such as variant interpretation or reporting are inefficient. To overcome this situation, we developed REDBot as an automated software package for accurate and direct generation of clinical diagnostic reports for prenatal and products of conception (POC) samples.

**Methods:**

We applied natural language process (NLP) methods for analyzing 30,235 in‐house historical clinical reports through active learning, and then, developed clinical knowledge bases, evidence‐based interpretation methods and reporting criteria to support the whole postdetection pipeline.

**Results:**

Of the 30,235 reports, we obtained 37,175 CNV‐paragraph pairs. For these pairs, the active learning approaches achieved a 0.9466 average F1‐score in sentence classification. The overall accuracy for variant classification was 95.7%, 95.2%, and 100.0% in retrospective, prospective, and clinical utility experiments, respectively.

**Conclusion:**

By integrating NLP methods in CNVs postdetection pipeline, REDBot is a robust and rapid tool with clinical utility for prenatal and POC diagnosis.

## Graphical abstract

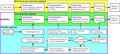

## 1 INTRODUCTION

In prenatal diagnosis, chromosomal aberrations, such as aneuploidies and copy number variation (CNV), are one of the important reasons for ultrasound structural abnormalities and products of conceptions (POC). CNVs are pervasive in human genome and account for a large fraction of the population diversity in humans (Girirajan, Campbell, & Eichler, 2011). Many CNVs located in specific genome regions also have clinical significance or have strong associations with well‐characterized genomic disorders, such as 22q11 deletion syndrome (Velocardiofacial/DiGeorge syndrome; Faas et al., 2010; Liao et al., 2014; Shaikh, 2017; Srebniak et al., 2012; Stankiewicz & Lupski, 2010).

Several methodologies are able to identify fetal CNVs in prenatal diagnosis, including noninvasive prenatal screening (NIPS) and invasive‐based methods such as karyotyping, chromosomal microarray (CMA), and next‐generation sequencing (NGS)‐based CNV sequencing (CNV‐seq; Brady, Ardui, & Vermeesch, 2013; Xie & Tammi, 2009). In recent years, some of these methods have rapidly become mature and have been recommended for clinical application (Oneda & Rauch, 2017). As a consequence, a growing number of pregnant women are electing for one of these diagnoses. For example, 94,085 women were enrolled for a large‐scale NIPS testing (Liang et al., 2019), and similar studies based on CNV‐seq or CMA platforms have been conducted (Muys et al., 2018; Wang et al., 2018). With the rapid improvements in CNV identification methods, complete clinical genetic diagnostic pipelines including postdetection analysis such as annotation and interpretation of CNVs, generation of diagnostic reports and genetic counseling approaches are in constant development. For post detection analysis, current approaches often rely on experts’ interpretation of CNVs: (1) CNVs are firstly classified as benign, likely benign, variants of uncertain significance (VOUS), likely pathogenic or pathogenic based on the American College of Medical Genetics (ACMG) guidelines (Richards et al., 2015; Riggs et al., 2019; Wang et al., 2018); (2) the contents of diagnostic reports for individuals are often manually generated by clinical laboratory geneticists. However, these approaches are time consuming and laborious, especially for some hospitals or companies where thousands of women may undergo prenatal diagnosis per month or the eligible geneticists are few. In practice, this inefficiency is mainly due to manual interpretation of CNVs and manual generation of diagnostic reports. From the interpretation perspective, despite the development of several tools for CNVs interpretation (Erikson, Deshpande, Kesavan, & Torkamani, 2015; Gai et al., 2010; Spector & Wiita, 2019; Vandeweyer, Reyniers, Wuyts, Rooms, & Kooy, 2011; Zhao & Zhao, 2013), none have proven the clinical utility in prenatal diagnosis. In addition, there are difficulties in dealing with VOUS CNVs due to limited investigative time as well as the lack of comprehensive genotype/phenotype databases (Brady et al., 2013, 2014; Levy & Wapner, 2018). Therefore, there is an urgent need in the field for an automated method that has the built in capacity to accurately and efficiently generate clinical diagnostic reports.

To address this issue, REDBot was developed to automatically generate clinical diagnostic reports based on CNVs called from the analysis of prenatal and POC samples. In this study, we demonstrate our new natural language process (NLP) methods and assess the performance of REDBot in a clinical diagnostic setting.

## 2 MATERIALS AND METHODS

### 2.1 Ethical compliance

The study protocol was approved by the local ethics committee at the authors’ affiliated institution. Patient consent was not required because of the retrospective nature of this study. Patient personal data were anonymized in the study.

### 2.2 Study design

**FIGURE 1:**
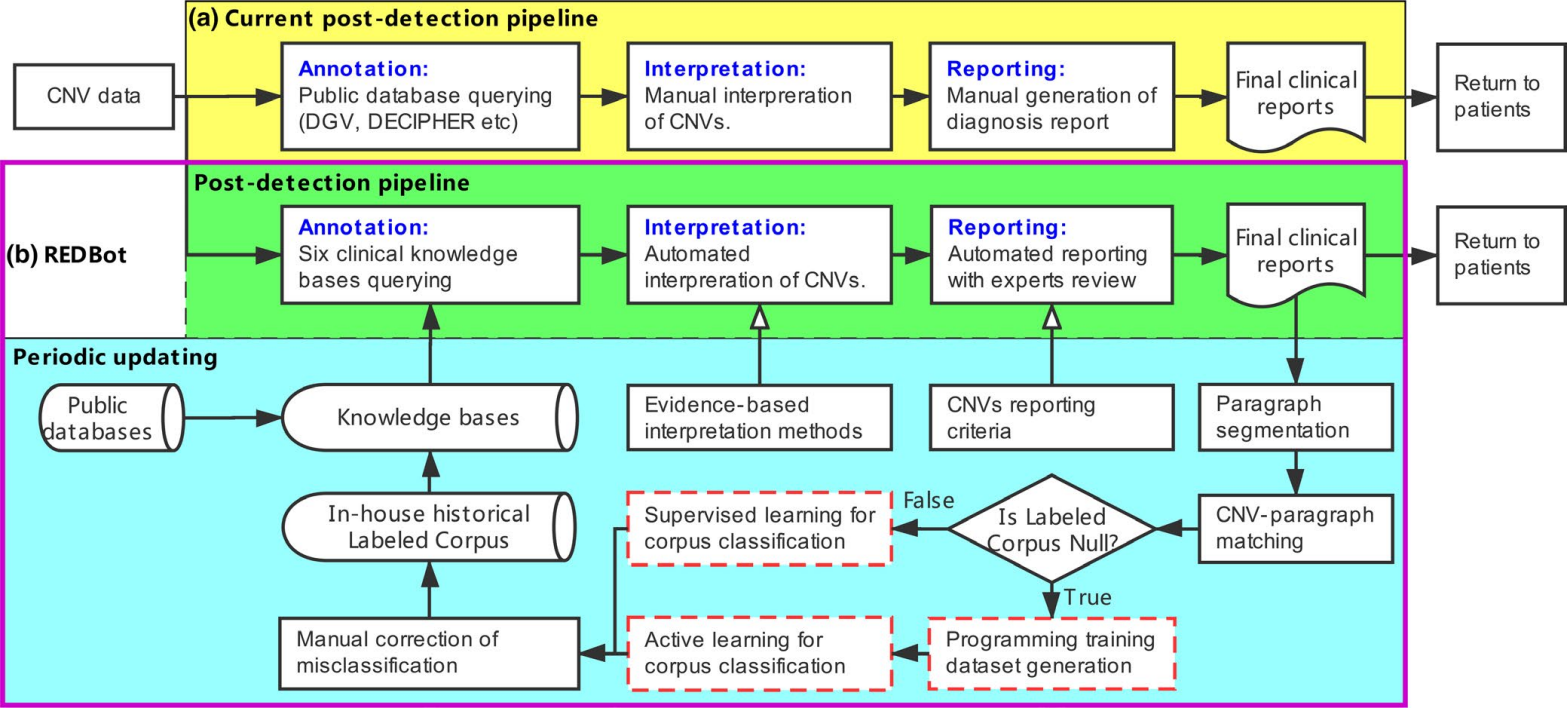
Current and REDBot approaches. (a) Current postdetection pipeline that includes annotation, interpretation, and reporting processes. It heavily relies on CNVs interpretation and diagnostic report generation by clinical laboratory geneticists. (b) The REDBot approaches. For postdetection pipeline, REDBot utilizes knowledge bases, evidence‐based interpretation methods, and CNVs reporting criteria to directly generate of clinical diagnostic reports. The periodic updating mainly focuses on applying NLP methods for analyzing in‐house historical clinical reports, and then, updating knowledge bases.

In light of the time consuming nature of current postdetection methods, REDBot was developed to provide an automated postdetection pipeline for reporting CNVs identified in prenatal and POC samples (Figure 1). In order to support the REDBot pipeline, knowledge bases, evidence‐based interpretation methods, and CNV reporting criteria were developed and periodically updated. For the updating process, NLP methods were applied to analyze in‐house historical clinical reports and eventually update or generate Labeled Corpus through supervised learning or active learning depending on whether a previous version of Labeled Corpus existed. In addition, the above Labeled Corpus as well as public databases were integrated into six knowledge bases for the annotation processes. Based on the annotation, interpretation, and reporting methods, REDBot was then able to directly generate clinical reports and these reports, which could be quickly reviewed by clinical experts before being sent out to patients.

### 2.3 Analysis of historical reports

**FIGURE 2:**
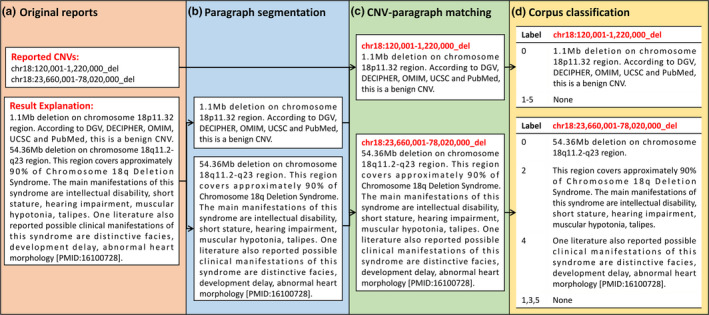
The three‐step approach for analyzing in‐house historical reports. (a) An example of a reported CNV region, and result explanation. In this historical report, the result explanation for CNVs was organized in one paragraph. (b) Paragraph segmentation: the original paragraph was segmented into two sub‐paragraphs based on Jieba for text segmentation, TF‐IDF for encoding and naive bayes model for classification. (c) CNV‐paragraph matching: scoring methods were developed to find best matches between CNVs and sub‐paragraphs. (d) Corpus classification: all sentences in sub‐paragraphs were classified into six categories through supervised learning or active learning. The result explanation in this example was originally in Chinese and we translated it to English.

The processes of analyzing historical reports required the application of NLP methods to convert original reports into in‐house historical Labeled Corpus. The original reports contained the identified CNVs and explanatory information in Chinese text. The result explanation was typically organized in one paragraph, however, multiple CNVs could be reported for a single sample. As such, this often led to one explanatory paragraph covering multiple CNVs. To deal with the above situation, a three‐step NLP approach was developed to address paragraph segmentation, CNV‐paragraph matching, and corpus classification. An example of our three‐step NLP approach is illustrated in Figure 2.

### 2.4 Paragraph segmentation

Paragraph segmentation refers to splitting the original paragraph into several sub‐paragraphs, where each sub‐paragraph refers to a result explanation of one specific CNV. The whole paragraph was first segmented by comma or period and we chose the first sentence of each report as the positive training data set. Other sentences from one‐CNV reports (reports that only contain one CNV with related description) were chosen as negative training data set. The testing dataset consisted of all historical clinical reports. For each sentence, we applied Jieba with parameter HMM = True for Chinese text segmentation and scikit‐learn with default setting for TF‐IDF encoding. We did not apply any rule on special tokens (e.g., “p11.32”), since these tokens may appear hundreds of times when analyzing more than 30,000 reports. After that, all sentences were classified as “begin” or “not begin” by the naive bayes model, where “begin” refers to the first sentence of CNV explanation. Finally, all sub‐paragraphs were generated according to the “begin” sentence.

### 2.5 CNV‐paragraph matching

CNV‐paragraph matching aims to find optimized matches between CNVs and sub‐paragraphs. The relation score between CNV *C_i_* and sub‐paragraph *S_j_* was calculated by:

$$\begin{array}{cc} \text{score}(C_{i},S_{j}) & =5\times \text{chr}\_\text{score}+2\times \text{type}\_\text{score}\\ +\text{cyto}\_\text{score}+\text{legenth\textbackslash{}\_score} \end{array}$$

where chr_score, type_score, cyto_score, and length_score were binary values determined by whether chromosome, variant type, cytoband, CNV length were identical in CNVs and sub‐paragraphs. For different type of scores, these values were calculated according to whether related keywords appear in sub‐paragraphs. For example, “deletion” or “duplication” refers to keyword of variant type score, “50 Mb” refers to a CNV length score and “p11.32” refers to a cytoband score. For a given CNV, the cytogenetic location (e.g. “p11.32”) was annotated according to UCSC hg19 cytoband file (http://hgdow​nload.cse.ucsc.edu/golde​nPath/​hg19/datab​ase/cytoB​and.txt.gz) and based on the CNV location. In addition, we applied empirical values as the weights for different type of scores based on our data set. For a given CNV *C*, the most related sub‐paragraph *S* was chosen by the following equation and we chose the first sub‐paragraph when multiple sub‐paragraphs had equal scores:

$$S=\arg\max(\text{score}\left(C,S_{i}\right))$$

### 2.6 Corpus classification

**FIGURE 3:**
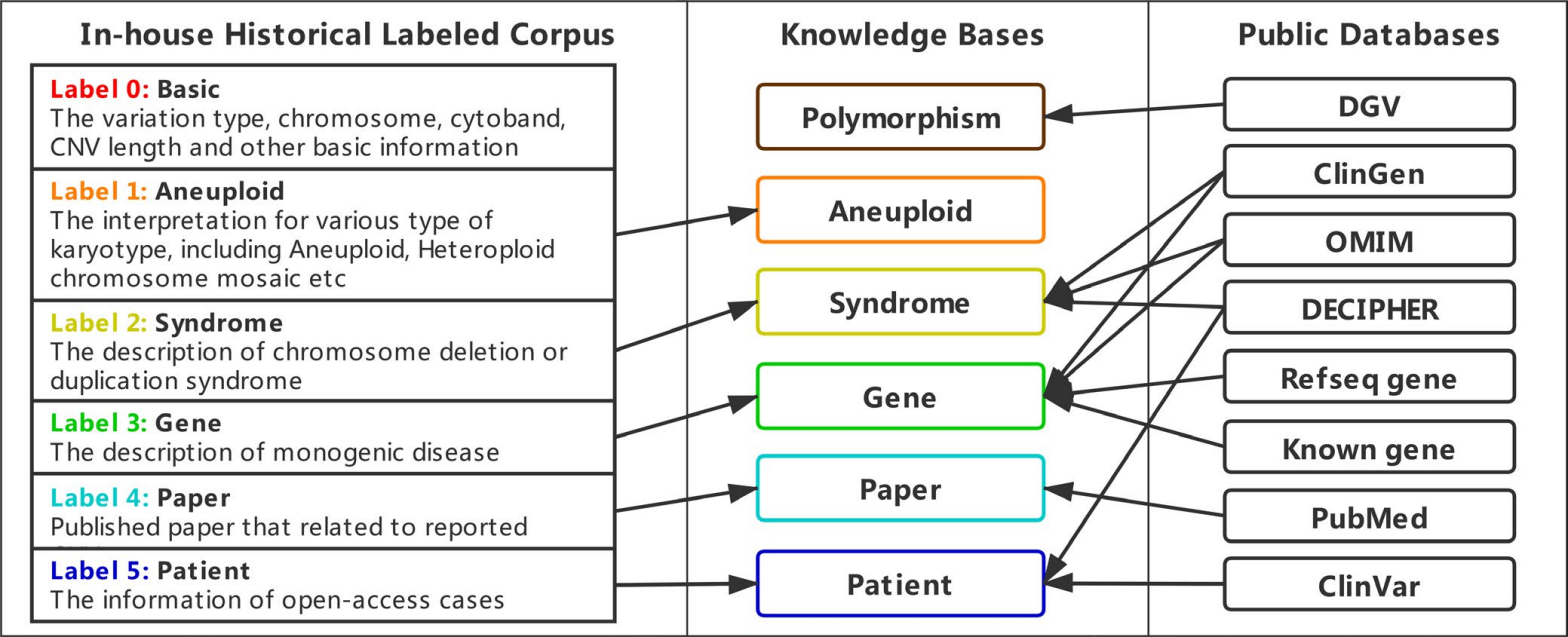
Relationship between in‐house historical Labeled Corpus, public databases and knowledge bases. In‐house historical Labeled Corpus demonstrated the six categories in corpus classification. Public databases mainly include OMIM (Hamosh, Scott, Amberger, Bocchini, & Mckusick, 2005), DECIPHER (Swaminathan et al., 2012), ClinGen (Kirkpatrick, Riggs, Azzariti, Miller, & Faucett, 2015), Refseq gene (Pruitt, Tatusova, & Maglott, 2005), Known gene (Hsu et al., 2006), Clinvar (Landrum et al., 2016), DGV (Macdonald, Ziman, Yuen, Feuk, & Scherer, 2014), PubMed etc. Knowledge bases described six knowledge bases in REDBot.

Corpus classification refers to applying sentence‐level classification methods to classify all sentences into six categories, namely, Basic, Aneuploid, Syndrome, Gene, Paper, and Patient (Figure 3) and these categories were integrated into Labeled Corpus. For the initial approach, when previous Labeled Corpus did not exist, a pool‐based active learning strategy was applied for corpus classification. In order to select candidate sub‐paragraphs for labeling, we applied programming training data set generation methods to first generate an inaccurate label for each sentence. Then, the classification model was trained based on the inaccurate label and sub‐paragraphs were chosen for labeling by clinical laboratory geneticists if any three consecutive sentences were classified as three different categories and if the number of different categories were greater than three in one sub‐paragraph. The label for the final classification model was based on those labels from clinical laboratory geneticists as well as from programming training dataset generation. We also applied a supervised learning approach if a previous Labeled Corpus existed, where we used them as a training data set, and new sub‐paragraphs as a testing data set. The final classification results were further evaluated by clinical laboratory geneticists in order to improve quality of in‐house historical Labeled Corpus.

For generation of programming training data sets, we applied a keyword string matching method to produce the inaccurate label for sentences. For example, if a key‐word such as “literature” was in one sentence, this sentence would be classified as Paper. For the classification model, GloVe, a global log‐bilinear regression model was firstly applied to pretrain 200 dimension token‐level vectors (Pennington, Socher, & Manning, 2014), and the vectors were trained on Chinese Wikipedia, in‐house historical reports and Chinese Human Phenotype Ontology (CHPO) database. Next, sentence vectors were represented by the average of token‐level vectors, and then, a bidirectional Long Short‐Term Memory with Conditional Random Field (biLSTM + CRF) model was used for classification. This model was applied in both active learning and supervised learning and was trained with following parameter: batch size = 64, num_epochs = 50, dropout rate = 0.1, learning rate = 0.001, max sequence length = 100, num_units = 200, optimizer = Adam.

### 2.7 Clinical CNV knowledge bases

Knowledge bases in REDBot were designed for providing structured information for clinical laboratory geneticists and improving annotation efficiency. We combined information from both public databases as well as in‐house historical Labeled Corpus to build six knowledge bases, namely, Polymorphism, Aneuploid, Syndrome, Gene, Paper, and Patient. The relationship of these databases is outlined in Figure 3. Importantly, knowledge bases only contain evidences rather than variant classification at a given point in time. Hence, for identical CNVs, variant classification in historical reports did not directly affect current variant classification.

### 2.8 Variant classification

**FIGURE 4:**
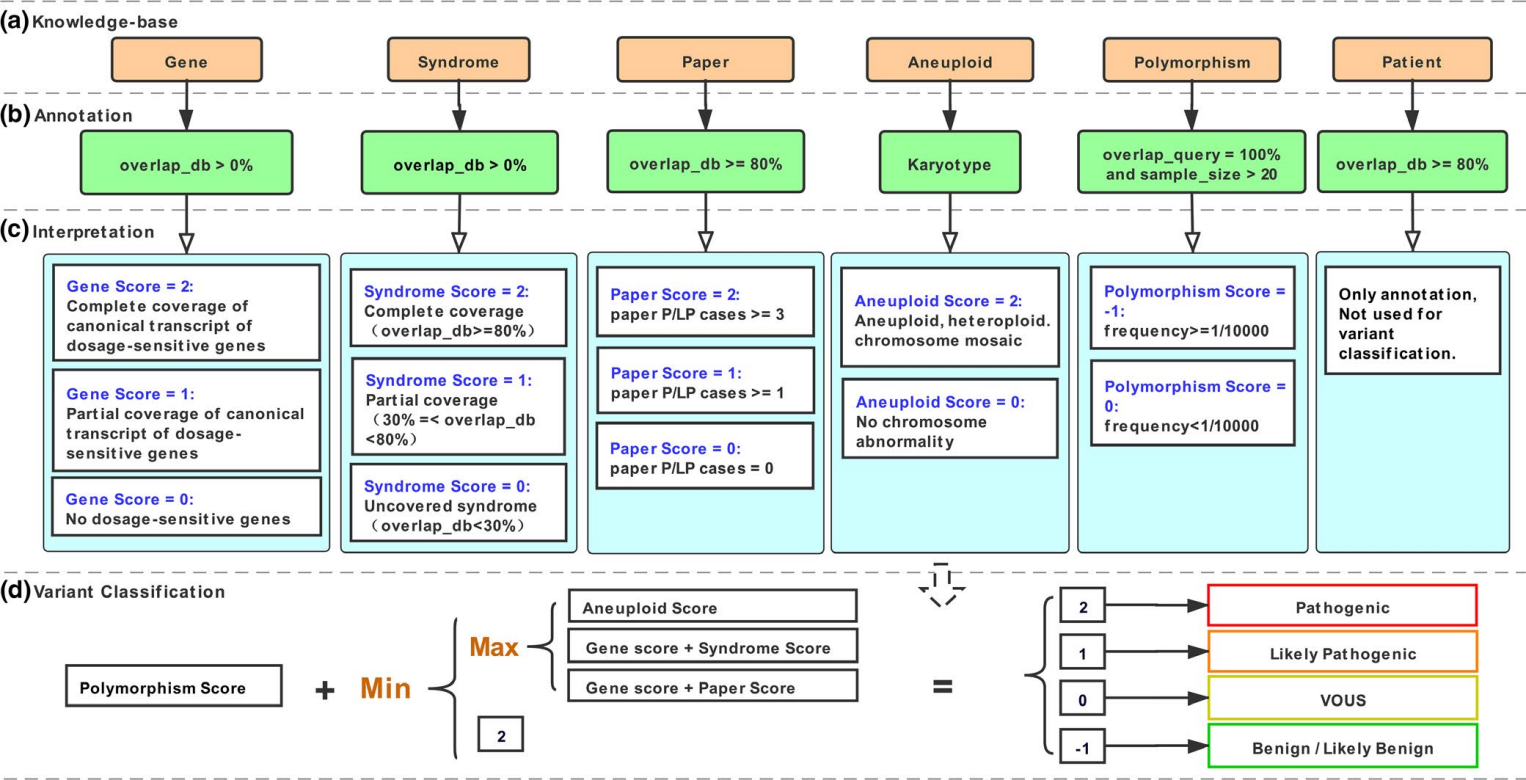
Algorithm of annotation, interpretation, and variant classification in REDBot. (a) The Knowledge‐based layer demonstrated six knowledge bases. (b) The Annotation layer gave the rule of annotation and all annotated information that can be further reviewed by clinical laboratory geneticists. (c) The Interpretation layer refers to evidence‐based methods for evidence scores calculation. (d) The Variant Classification layer shows the equation between evidence scores and final classification. Definitions: overlap_db, the overlap length over the region length in knowledge bases; overlap_query: the overlap length over the queried CNVs length; sample_size: the sample size of each study in DGV database; frequency: allele frequency of CNVs; paper P/LP cases: total number of reported cases (only count one if several cases in one trio) in published literature.

The majority of classification criteria incorporated into REDBot (Figure 4) was based on 2011 ACMG guidelines (Kearney, Thorland, Brown, Quintero‐Rivera, & South, 2011). The annotation section takes the CNVs region(s), gender, and variation type (homozygous deletion, heterozygous deletion, duplication, or triplication) as input data. Information from six knowledge bases that generally represent all aspects of CNVs were retrieved based on filters in Figure 4b. The interpretation section refers to calculating evidence scores for CNVs from the knowledge bases, using different calculation methods (Figure 4c). The variant classification section refers to making a final classification based on the equation shown in Figure 4d. In addition, CNVs with incomplete penetrance (e.g., some recurrent regions) did not influence the variant classification.

### 2.9 Generation of clinical diagnostic reports

Based on the variant classification results, REDBot automatically generates diagnostic reports for review by clinical laboratory geneticists. For specific types of aneuploidy, heteroploidy, and chromosome mosaicism, reports were generated by applying the description from the Aneuploid knowledge base. For CNVs, the reports were composed by (1) basic description: the information about cytogenetic location, variation type, and CNV length; (2) special explanation: a clear statement of related syndrome, gene dosage sensitivity, or cases followed by clinical significance and special considerations. In order to generate the above special explanation, we applied sentences (corpus) from knowledge bases for pCNVs and a template sentence “according to DGV, DECIPHER, OMIM, UCSC and PubMed databases and ACMG guidelines, this is a benign (or VOUS) CNV” for benign (or VOUS) CNVs.

### 2.10 Assessment and evaluation

Several data sets were selected for development and assessment of REDBot. In general, the performance of REDBot was investigated in four ways:

1. NLP for analyzing historical reports: 11,611 prenatal and 18,624 POC diagnostic reports between July 2015 and February 2019 were selected for knowledge bases development. We estimated the performance of NLP approaches, namely, paragraph segmentation, CNV‐paragraph matching, and corpus classification.
2. Retrospective experiments: 3,372 prenatal and 1,679 POC diagnostic reports (5,051 CNVs in total) between January 2018 and December 2018 were selected for performance evaluation of variant classification. These reports were also selected for knowledge bases development.
3. Prospective experiments: This approach was basically same as for retrospective experiments, except that diagnostic reports for 239 prenatal and 185 POC samples (580 CNVs in total) between March 2019 and April 2019 were not included in knowledge bases development.
4. Clinical utility: Only REDBot was applied for variants classification and reports generation in a randomly selected set of 100 prenatal and 100 POC samples (250 CNVs, aneuploid, etc. in total) in July 2019, and then, the results were estimated by clinical laboratory geneticists from perspectives of both variant classification and reporting.

All historical clinical reports are in Chinese and CNVs with minimum length of 100 Kb were identified on the CNV‐seq platform developed by Berry Genomics Corporation, Beijing, China (Liang et al., 2014). The hg19 sequence (GCA_000001405.1) from the GRCh37 Genome Reference Consortium was used as reference for calling CNVs. The authenticity of called CNVs was reviewed by a group of genetic experts in the company.

## 3 RESULTS

### 3.1 Information in historical report

The quality of in‐house Labeled Corpus is essential not only in knowledge base generation, but also the whole postdetection REDBot pipeline. Therefore, the results from NLP approaches were estimated and corrected manually before downstream analysis.

We first estimated the performance of paragraph segmentation combined with CNV‐paragraph matching, which achieved an overall accuracy of 99.22%. In detail, 5,646 out of 30,235 reports contain more than one CNV, aneuploidy or chromosome mosaicism. By excluding 44 reports with incorrect results, a total number of 37,175 sub‐paragraphs were eventually obtained for corpus classification. In terms of corpus classification, each sentence was classified as a specific class of corpus, namely, Basic, Aneuploid, Syndrome, Gene, Paper, and Patient. In reality, the performance on Syndrome, Gene, and Paper are extremely important since it influences both variant classification as well as CNV description in reports. The class of Aneuploid always indicates pathogenic outcomes. In addition, the majority of records from Patient knowledge base are from open‐access databases and thus are only used in annotation. Finally, the class of Basic does not directly contribute to variant classification, since it only refers to key indices such as cytogenetic location, CNV size, and copy‐number state.

We then estimated the performance of programming training data set generation, which applies to producing inaccurate labels for corpus classification based on string matches, and thus, the statistic metrics can also be regarded as a baseline for active learning. This approach achieved an average F1‐score of 0.8365, however, but the F1‐scores for Syndrome, Gene, and Paper were only 0.7868, 0.5041, and 0.7758, respectively (Table 1). By applying active learning through biLSTM + CRF, we labeled only 815 sub‐paragraphs among 37,175, and these values improved to 0.9381, 0.8645, and 0.9195. On the one hand, in the actual reports, the indication of specific syndrome, gene, or literature is often followed by a description of clinical symptom(s), phenotype, or any other recommendations. Therefore, it is unlikely to have three different labels for three consecutive sentences. However, due to linguistic diversity, this issue did occur in generating programming training data sets. Hence, in active learning, we aimed to identify and label those sub‐paragraphs to improve the overall performance of classification. On the other hand, due to less diversity of related description, the accuracy of Basic, Aneuploid, and Patient was higher than that of Syndrome, Gene and Paper. In addition, sentences of Basic or Patient were often at the beginning or at the end of sub‐paragraphs, respectively, further improving the performance of these classes. Finally, comparing different classification models in the active learning strategy, the biLSTM + CRF outperformed NB + CRF for all classes. This probably indicates that the semantics of continuous sentences play an important role in corpus classification, since biLSTM is a powerful tool for modeling context relationship. After manual correction of incorrect classification, we eventually obtained 15169, 17575, 2260, 1304, 3173, and 1073 records for Basic, Aneuploid, Syndrome, Gene, Paper, and Patient, respectively. These records were further applied in developing knowledge bases.

**TABLE 1:** Performance of active learning in corpus classification.

Label	Programming training data set			Active learning (NB + CRF)			Active learning (biLSTM + CRF)		
	Precision	Recall	F1‐score	Precision	Recall	F1‐score	Precision	Recall	F1‐score
Basic	0.9906	0.9734	0.9819	0.9883	0.9654	0.9767	**0.9933**	**0.9735**	**0.9833**
Aneuploid	0.9781	0.9780	0.9781	0.9740	0.9771	0.9755	**0.9782**	**0.9782**	**0.9782**
Syndrome	0.6881	0.9186	0.7868	0.8067	0.8624	0.8336	**0.9248**	**0.9518**	**0.9381**
Gene	0.4092	0.6564	0.5041	0.6207	0.8044	0.7007	**0.7973**	**0.9440**	**0.8645**
Paper	0.7702	0.7816	0.7758	0.7857	0.7813	0.7835	**0.9207**	**0.9184**	**0.9195**
Patient	0.9925	0.9925	0.9925	0.9748	0.9748	0.9748	**0.9953**	**0.9963**	**0.9958**
Average	0.8048	0.8834	0.8365	0.8584	0.8942	0.8741	**0.9349**	**0.9604**	**0.9466**

### 3.2 Accuracy of variant classification in retrospective and prospective experiments

The performance of variant classification using the REDBot pipeline was independently evaluated by clinical laboratory geneticists, following the five‐tier standard terminology system from ACMG guidelines (Kearney et al., 2011; Richards et al., 2015; Riggs et al., 2019). In addition, for reporting criteria, we used benign to represent both benign and likely benign from ACMG guidelines and this was clearly indicated in reports and laboratory reporting protocols. The classification from clinical laboratory geneticists was regarded as the gold standards in order to judge the performance of REDBot. The accuracy was calculated through two approaches, first by, comparing whether CNVs were classified as pCNVs (pathogenic or likely pathogenic CNVs) and second by comparing the exact variant classification.

**FIGURE 5:**
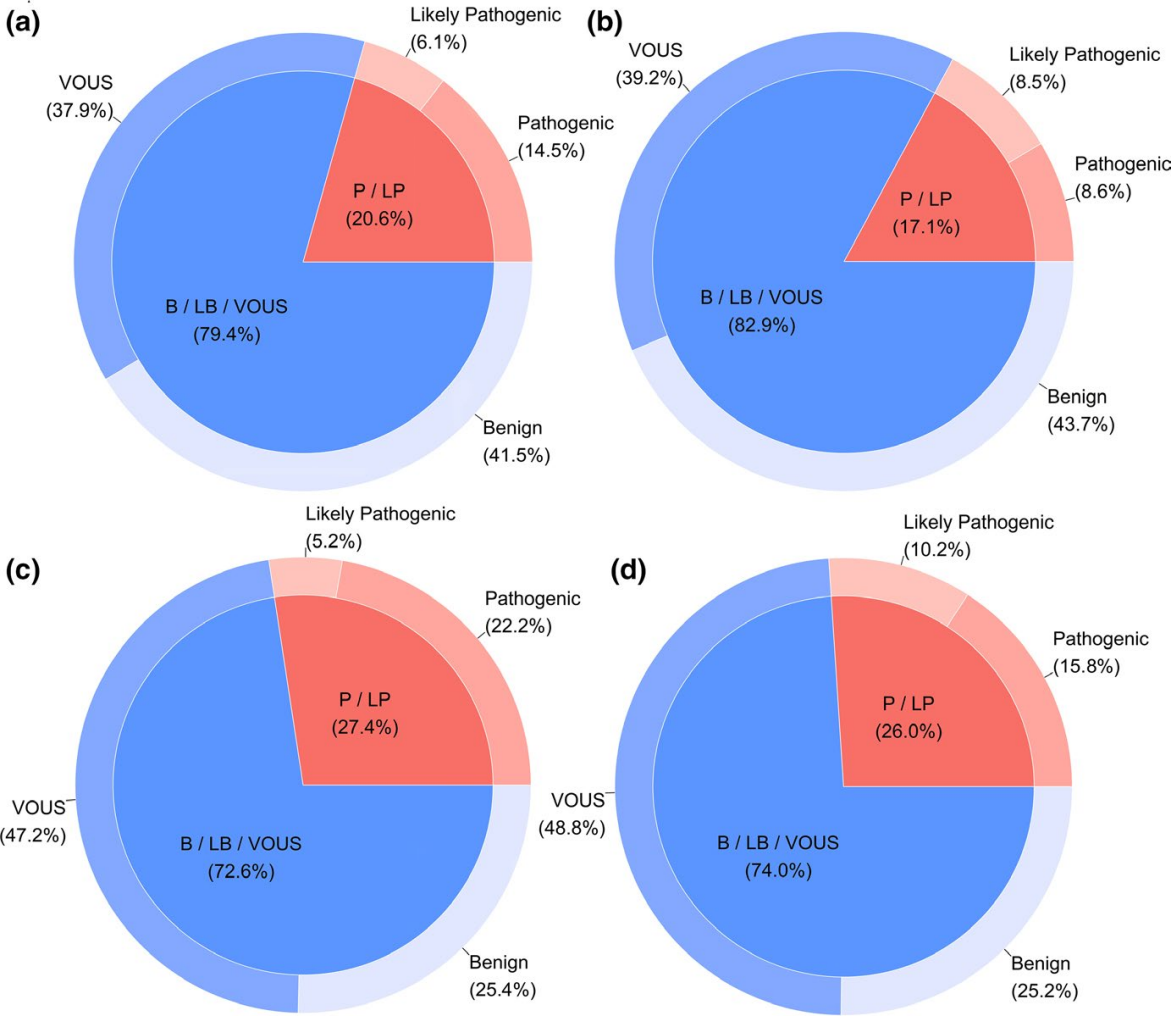
The proportion of variant classification from REDBot and clinical laboratory geneticists. The inner pie chart demonstrated proportion of pCNVs and the outer one represented proportion of exact classification. (a) Retrospective experiment results from REDBot; (b) Retrospective experiment results from clinical laboratory geneticists; (c) Prospective experiment results from REDBot; (d) Prospective experiment results from clinical laboratory geneticists.

For the first approach, among the CNVs in retrospective (n = 5051) and prospective (n = 580) experiments respectively, the overall accuracy achieved was 95.7% and 95.2% (Table 2), suggesting that REDBot was capable of distinguishing pCNVs. In addition, comparing results with clinical laboratory geneticists, there were higher negative predictive values (NPVs) than positive predictive values (PPVs) indicating that REDBot calls a higher proportion of pCNVs. The overall increased in pCNV positive rate calls was 3.5% for retrospective and 1.4% for prospective experiments (Figure 5). For the second approach, we also obtained high accuracies of 86.6% and 85.2%, indicating the majority of CNVs were correctly classified. In addition, the proportion of different CNV categories in the retrospective and prospective experiments was also similar.

**TABLE 2:** The performance statistics of variant classification in REDBot.

Statistics	Retrospective experiment	Prospective experiment	Clinical utility
Total	5051	580	250
TP	846	141	140
TN	3988	411	110
FP/FPR	197/4.7%	18/4.2%	0/0.0%
FN/FNR	20/2.3%	10/6.6%	0/0.0%
PPV	81.1%	88.7%	100%
NPV	99.5%	97.6%	100%
Sensitivity	97.7%	93.4%	100%
Specificity	95.3%	95.8%	100%
Accuracy	95.7%	95.2%	100%
Kappa	86.0%	87.7%	100%

Abbreviation: FN, false negative; FNR, false negative rate; FP, false positive; FPR, false positive rate; NPV, negative predictive value; PPV, positive predictive value; TN, true negative; TP, true positive.

The primary reason for incorrect CNV classifications was the unbalanced sensitivity between REDBot and clinical laboratory geneticists, whereby REDBot sometimes predicted likely pathogenic as pathogenic or VOUS as likely pathogenic. Therefore, in clinical practice, REDBot may generate an increased yield of positive results for human reviewers, Conversely, REDBot may reduce false negative results since no pathogenic or likely pathogenic CNVs were predicted to be benign. In further analyzes, we found that REDBot provides more pathogenic evidence according to ClinGen Dosage Sensitivity Map. In addition, the paper score might be higher in REDBot due to a higher number of annotated cases.

Comparing the performance of retrospective and prospective experiments, the overall accuracy of CNV interpretation in the former one was slightly higher. This was attributed to clinical laboratory geneticists who were finding additional cases in the published literature, which were not included in current version of paper knowledge base. However, in general, the similarity of the performance indicated that REDBot did not have a tendency to “over‐fit” the data into each category. Hence, REDBot proved to be robust for generating accurate prenatal and POC diagnostic reports. Details of the results of both retrospective and prospective experiments are documented in Table S1.

### 3.3 Experiments to assess practice clinical utility

In the randomly selected 200 samples, the pathogenic classification by REDBot was completely consistent with clinical laboratory geneticists (Table 2). For practice clinical utility, the performance improved, since 38% of reported chromosome abnormalities associated with aneuploidy, heteroploidy, or chromosomal mosaicism (either duplication or deletion events of whole chromosomes), and thus, were pathogenic. There were small differences in the illustrated parts. For benign or likely benign CNVs reported by REDBot, clinical laboratory geneticists did not generally report them, mainly because of the fear of misleading clinicians and patients. To sum up, the analysis and illustrations by REDBot were highly consistent with geneticists working in the clinical diagnostic field. Full details of the results of practice clinical utility experiments are given in Table S2.

### 3.4 Implementation of REDBot

REDBot was developed to facilitate the whole postdetection pipeline and improve the practice utility for prenatal and POC diagnosis. We wrote scripts to automatically analyze in‐house historical reports and public databases to generate six clinical knowledge bases. Next, the annotation, interpretation, and report generation algorithms were implemented in C++, and static link libraries were constructed to facilitate other developers such as front‐end developers. We also evaluated the efficiency of REDBot, and demonstrated that on average 31.54 reports can be generated within 1 second on a personal computer.

## 4 DISCUSSION

Testing for fetal genome‐wide pCNVs in prenatal diagnosis is now well accepted by clinicians and patients. In China, with a population of 1.4 billion and 16 million annual births, the total number of individuals with chromosomal abnormalities was estimated to be over 10 million. Therefore, prenatal diagnosis plays an essential role in prevention by reducing the burden of chromosome diseases (Cram & Zhou, 2016). From a technical point of view, there have been significant improvements in CNV identification methods using both sequencing‐ and array‐based platforms, which can provide reliable and accurate detection (Chen et al., 2017; Venkatraman & Olshen, 2007). In addition, with decreasing of sequencing costs combined with the fast growth of the Chinese economy, genetic diagnosis is now more affordable for the general population. Currently, genetic services for identification of genome‐wide pCNVs is typically provided by major hospital laboratories and by commercial companies. Accordingly, there is an increasing workload for the laboratory and clinical geneticists to deliver reports to the referring clinician.

Given this increasing clinical demand for first‐line CNV detection, application of REDBot will potentially help to solve several current major issues in the genetic service pipeline, including inconsistent or incorrect results of CNV annotation and interpretation from both commercial companies and medical institutions and help reduce the time taken for qualified clinical laboratory geneticists to properly interpret the clinical significance of CNVs. Further, there are additional difficulties for clinical geneticists to review or reanalyze CNVs based on phenotype due to poor communication between commercial companies and medical institutions. Despite the fact that pipelines for laboratory experiments and bioinformatics approaches for CNVs analysis are completed automated, current CNVs interpretation methods and the generation of reports is time consuming and, in some cases, can comprise management of the pregnancy. Hence, an accurate, efficient, and automated postdetection pipeline like REDBot will help to increase the overall efficiency of prenatal diagnosis.

Based on a collection of 30,235 historical reports, REDBot displayed new approaches for dealing with the postdetection dilemmas of CNVs found in prenatal and POC samples. We presented NLP methods for analyzing historical reports and eventually developed knowledge bases for annotation. Therefore, CNV‐related information can be easily investigated if the reported CNVs that share similar genome coordinates to previously reported CNVs. The accuracy of variant classification is extremely important, since this will influence the parental decision‐making on pregnancy termination. In regard to accuracy, we estimated the performance of variant classification for over 5,000 CNVs through retrospective, prospective, and clinical utility experiments. The 95% accuracy achieved (Table 2) highlights the stability and reliability of REDBot. Thus, REDBot should be a good assistant for clinical experts by improving their overall work efficiency and decreasing reporting time. In the future, with the growing number of historical reports available in different clinical settings, REDBot can be further trained to generate more comprehensive clinical reports, allowing clinicians to make better decisions for the management of at risk pregnancies.

The classification or interpretation in REDBot relies on each CNV alone. In addition, REDBot also assumed all input CNVs are true positive for individuals regardless of sample type, and generated consistent results for identical CNVs. Due to complication of practical clinical settings, such as CNVs resulted from structural rearrangements (e.g., unbalanced translocation), REDBot should be applied under the supervision of laboratory geneticists or clinical experts.

In the immediate future, further improvement of REDBot will be focused on three aspects. First, with the rapid development of deep learning and natural language generation (NLG) technology (Gatt & Krahmer, 2017), it might be possible to apply these methods for improving personalized diagnostic reports. Second, the application of REDBot can also be expended to Whole Exome Sequencing (WES) or Whole Genome Sequencing (WGS)‐based diagnosis by integration of the new ACMG interpretation and reporting guidelines (Riggs et al., 2019). Third, it is beneficial for REDBot to train the model based on reports in different languages as well as from different medical institutions, in order to become a universally useful tool for the whole postdetection pipeline. Finally, with the increasing number of genetic diagnosis reports, REDBot will be continuously updated and improved in order to deliver more comprehensive and personalized genetic reports to clinicians.

## CONFLICT OF INTEREST

Mengmeng Liu, Yunshan Zhong, Erhong Liu, Yu Zhang, Feng Tian, and David S. Cram are employees of Berry Genomics Corporation. With the exception of Feng Tian, none of the authors hold any stocks or bonds in the company. The other authors declare no conflicts of interest.

## AUTHOR CONTRIBUTION

Fuli Yu contributed to the conception of the study and manuscript. Mengmeng Liu performed the data analyses and developed REDBot software. Yunshan Zhong contributed to the NLP algorithm. Hongqian Liu evaluated REDBot in practice clinical utility. Desheng Liang, Erhong Liu, Feng Tian, and Lingqian Wu contributed to evidence‐based variant classification method. Desheng Liang additionally reviewed clinical reports and manuscript and Erhong Liu also contributed to prospective experiment. Yu zhang offered suggestion on manuscript and software development and prepared responses for reviewer(s). Feng Tian provided historical reports for this study. David S. Cram edited the manuscript to improve grammar and meaning. Other authors contributed to review clinical reports and provide advice.

## Supporting information

Table S1

Table S2

## Data Availability

The data that support the findings of this study are available from the corresponding author upon reasonable request.
